# Book Reviews

**Published:** 1827-09

**Authors:** 


### CRITICAL ANALYSES.

Quæ laudanda forent, et quæ culpanda, vicissim

Illa, prius, cretâ; mox hæc, carbone, *notamus.*—PERSIUS.

*Elements of Physics, or Natural Philosophy, General and Medical, explained independently of Technical Mathematics.* By N. ARNOTT, M.D. of the Royal College of Physicians.—8vo. pp. xxxv. 611. Underwood, London, 1827.

ALTHOUGH the term "physical" still remains in the title of our Journal, yet it has become so entirely a "medical" periodical, that we scarcely regard works on natural philosophy as coming within the scope of our critical labours. But the volume before us is so peculiar in itself, bears so much upon medical topics, and is withal so excellent, that we should regard ourselves as wanting in attention to the interests of our readers if we neglected to introduce it to their notice, and to recommend it very strongly to their perusal. To medical students especially it must prove highly useful, and ought to be on the shelves of all those who are desirous of acquiring not merely a limited and partial knowledge of particular facts, but who would understand the great laws which regulate the actions of the human body in common with all other matter. The application of physics to the objects of our profession is indeed very extensive, and embraces some of the most important branches of natural philosophy. The limbs, for example, are all dependent on the general principles of *mechanics;* the bones, joints, muscles, and tendons, are but so many levers, hinges, and pulleys;—the heart and blood-vessels obey the laws of *hydraulics;* the lungs of *pneumatics;* the eye of *optics;* and the ear of *acoustics.* These considerations ought to have weight, independent of a knowledge of natural philsophy being an essential part of a liberal education, such as every practitioner ought to possess, and such as the multiplied facilities of acquiring knowledge place within the reach of every one.

It is not our object, of course, to enter into the general details of a work of this nature; but we shall select some parts which are calculated to show the connexion between the contents of the volume and those subjects which more peculiarly belong to our profession. We hope, too, that the specimens we give will induce our readers to seek for that connected and satisfactory information which can only be obtained from a perusal of the whole.

Under the head of Animal and Medical Mechanics, we have some interesting remarks on the mechanism of the skeleton, particularly of the skull; a part of the subject also well illustrated by Mr. C. Bell, in his anatomical writings, and still more in his oral lectures. Next, perhaps scarcely second in interest as a piece of mechanism, is the spine, the strength, elasticity, and flexibility of which are severally pointed out; considerations which naturally lead to the following remarks connected with spinal distortions:

"To the well-being of the higher classes of animals, exercise of their various parts is as necessary as nourishment, and, if it be withheld by any cause during the period of growth, the body is often crippled permanently. The overflow of life and energy which nature has given to young creatures to prompt them to useful exertion, is seen in the ever-changing occupation of a child, in the quick succession of its ideas, in its jumping and skipping, and using all sorts of round-about action that may expend muscular energy, instead of seeking, as in after-life, to accomplish its ends in the shortest ways: and the same law is illustrated among the inferior animals, by the play of kittens, puppies, lambs, &c.

"Strongly as nature has thus expressed herself upon the important subject of exercise among the young, tyrant fashion, with a usual perversion of common sense, has of late times, in England, formed a school discipline for young women of the higher classes, which wars directly with nature's dictate; and the consequences have been such, that a stranger arriving here from China might almost suppose it the design to make crooked and weak spines by our school discipline, as it is the design in China to make little feet by the iron shoe. The result is the more striking, because the brothers of the female victims, and who of course have similar constitutions, are robust, healthy, and well formed. A *peasant girl* is allowed to obey her natural feeling when her spirits are buoyant, and at proper times may dance, and skip, and run, until healthy exhaustion ask that repose, which is equally allowed; and she thus grows up strong and straight: but the *young lady* is receiving constant admonition to curb all propensity to such vulgar activity, and often, just as she subdues nature, she receives the praise of being *well-bred*. Her multifarious studies come powerfully in aid of the admonition, by fixing her for many hours every day to sedentary employment. This adoption of sedentary habits is not only hurtful, by preventing the natural extent and variety of exercise, and thereby weakening the whole body, but is rendered particularly injurious to the back, by the manner in which the sitting is usually performed. It would be accounted great cruelty to make a delicate young creature stand all day, because the legs would tire; but this very cruelty is almost in constant operation against the back, as if backs would not tire as well as legs. When a girl is allowed to sit down because she has been long standing, great care is taken that the muscles of the back, which still remain in action as she sits, shall not be at all relieved; for, from the idea that it is ungraceful to loll, she is either put upon a stool which has no back at all, or upon a very narrow chair with a perpendicular back. The stool relieves her spine more than the chair, because it allows of bending in different ways, so as to rest the different sets of muscles alternately; but the chair forces her to keep the spine quite upright and nearly unmoved. The consequence soon is, that, being first weakened generally by sedentary habits, and the back being still farther weakened by excessive fatigue, the spine gives way in some part and bends, and the curvature becomes permanent. In this bending the spine is sometimes partially rotated, so as to show from behind that waving profile which should only be seen from the side. At other times, the vertebræ and intervening substance merely become thinner on one side, from the continued pressure and weakness. When a bend takes place in one situation, it immediately occasions an opposite bend above or below it, to bring the centre of gravity of the upper parts directly over the base again; and hence the curve becomes double, like an Italic *f*.

"When the inclination of the back has once begun, it is very soon increased by the means used to cure it. Strong stiff stays are put on, to support the back, as it is said, but which in reality, by preventing those muscles from acting which are intended by nature as the supports, cause them to lose their strength, and, when the stays are withdrawn, the body can no longer support itself. Longer sittings in the narrow upright chair are then recommended, and sometimes the back is forcibly stretched by pulleys, or the patient is kept all day and night lying on an inclined board, and losing her health, &c. &c. The only things forgotten are to give proper exercise in the air, and to let the child rest when she is not taking such exercise. The prejudice had at last grown up, that strong stays should be put upon children very early to prevent the first beginnings of the mischief, and that a child should always be made to sit on the straight-backed chair, or to lie on the hard plane: and it is probable that, if these cures and preventives had been adopted as universally and strictly as many deemed them necessary, we should not have in England a young lady whose back would be straight or strong enough to bear the weight of her shoulders and head. It would disgust us to see the attempt made to improve the strength and shape of a young race-horse or greyhound, by binding tight splints or stays round its beautiful young body, and then tying it up in a stall; but this is the kind of absurdity and cruelty so commonly practised in this country towards what may well be called the most faultless of created things.

"A pernicious prejudice, with respect to this curvature or distortion of the spine, long existed,—viz. that it was a scrofulous affection; and many mothers hid it from those about them, and sought remedy from quacks far from home. Indeed, until within a few years, it was altogether the province of some irregular members of the profession to manage spine diseases, and it became to them a rich source of wealth; for many of their remedies being calculated to prolong the evil, they did not soon lose their patients.

"A man of considerable note in this class lately pronounced a case of crooked spine, which fell into his hands, to be a dislocation of the whole middle part of it; and, in the published account of the treatment which he said had effected a cure, he had the folly to assert that each day, by his various operations, he replaced one vertebra of the number dislocated, until at last the whole were restored. He thus accused himself of having torn each vertebra from those about it, and of treating a dislocated spine as that man would treat a dislocated arm, who should break the bone into pieces of an inch, and replace these successively, instead of letting the whole return as a single piece, in the common way.

"The practice in spine cases, however, has now fallen into the hands of the informed profession, and science having detected the true causes of the evil, its frequency is already diminished. It has been shown that nothing is easier than to prevent it, and that the best cures are those conducted on the general principles of improving the health of the patient, and of using exercises which directly strengthen the affected part.

"Some might expect here a long description of machines employed in the treatment of such affections; but fortunately the list of the only ones which are useful or safe is very short:—a sofa to rest upon, and choice of pleasant means of taking exercise,—such as the skipping-rope, shuttlecock, dumb-bells, a rope-ladder to climb, a winch to turn, &c.; and sometimes, where it is much desired that the young lady should continue her lessons of music in a sitting attitude, a chair may be used, having an overhanging canopy or crane, from which straps descend to support the head and shoulders, while proper weights, fixed to cords from these straps, and acting over pulleys, give the required support. The author has had a small light crane of wood made, which answers the purpose well, and may be attached to a common chair. It would be out of place here to detail those particulars of constitutional treatment which so usefully aid the effects of suitable exercise." (P. 198.)

The mechanism of the other parts is afterwards described, and the necessity of a constant attention to the laws of mechanics in the treatment of fractures and dislocations, and the great and useless torment which a neglect of them frequently gives rise to, is strikingly illustrated.

On the mechanism of various surgical instruments, we have the following observations:

"*Midwifery forceps.*—The blades beyond the joint or fulcrum being longer than the handles, the pressure on the child's head is less than that made by the hand, but it should always be kept present to the mind of him who uses the instrument. The substitute for the forceps proposed by the author, and described in the section on Pneumatics, it is hoped, will save infant life and maternal suffering, in many cases where unpractised hands might try the forceps in vain.

"The *vectis*, or lever used instead of the forceps to advance the child's head in parturition, is a very dangerous instrument in unskilful hands. In fact, whenever it is used as a lever, in the most common sense of the word, it is a piece of unskilful cruelty. Suppose any part of the pelvis to be made the fulcrum of this lever, the soft parts between the bone and the instrument are bruised with the whole force of the hand, and with twice or three times as much if the resistance be nearer to the fulcrum than to the hand. The instrument can only be safely used, by the operator making one of his hands the fulcrum, while the other is used as the power, or by his using one hand so as to answer both purposes; and then there is a resemblance between the action of the vectis and that of a hook.

"The *levator*, or lever for raising the broken and depressed portion of the skull in trepanning, has a fulcrum attached to it. Care should be taken to place this fulcrum where its pressure cannot be injurious.

"The *circular*, or *crown saw* of the trephine, should be turned or worked with a quick motion and gentle pressure, for the reason given at page 156, when describing cutting instruments. The object is thereby sooner and better attained, and the head of the patient is shaken much less.

"For the same reason, a light and quick motion of the saw should be used in dividing the bone in amputations: there is thus less jarring and an easier division.

"In using the *amputation knife*, also, the speed, neatness, and success of the operation, are all favoured by mixing the drawing or saw motion in the knife with the pressure towards the bone.

"These last observations are of a hundred similar which might be made to prove of what vital importance it is for a surgeon to have familiarity with the use of tools or instruments. Perhaps a person cannot acquire this better than by amusing himself with some work of carpentry. Manual dexterity, and a little readiness at mechanical contrivance, so frequently prove of importance to persons in all stations, that it is surprising why, in systems of general education, the matter has not received greater attention. If a handless or awkward man embrace the medical profession, and unfortunately attempt to practise surgery or midwifery, although he be possessed of brilliant intellect, he may fail in almost every thing which he attempts.

"The *tooth-key* is an instrument found in the hands of almost every body who pretends even to the lowest degree of skill in the healing art: and there is perhaps scarcely a day passing, in which teeth are not broken, and jaws splintered, and gums bruised even to sloughing, by the unskilful or awkward use of it. The common tooth-key may be regarded in the light of a wheel and axle, with the hand acting on two spokes of the wheel to move it, while the tooth is drawn to the axle by the claw, and is drawn out as the axle turns. The gum and alveolar process of the jaw form the support on which the axle rolls. The common errors of the operation of tooth-drawing by the key are these:—

"1st. Turning it towards that side where the adjoining teeth are too close for the moved tooth to pass, without either being broken itself or breaking one of them. Sometimes two teeth are thus drawn instead of one.

"2d. Neglecting the natural inclination of the tooth. By winding it round in the direction in which it already inclines, and in accordance with a bend which is generally found in the tooth itself, the operation is easy and safe; while, if drawn in the opposite way, it not unfrequently breaks or splinters the part of the jawbone in which it sticks.

"3d. If the tooth-claw is blunt, and its point slips high upon the tooth, it then acts almost directly across, and is very apt to break the tooth.

"4th. And unless the axle or fulcrum of the key be made to rest as evenly as possible on the gum, it will tear or very much injure the gum. It should bear or rest, if possible, over the part of the bone in which the tooth is set; for otherwise, as when a back tooth is drawn with the instrument resting on a part considerably anterior to it, the twist produced is painful, and there is danger of splintering. —A man whose study or reflection has suggested these remarks, and who then operates leisurely a few times on the dead subject, will be able to give instant and safe relief to most intense suffering. And it is hardly excusable in any medical man who may be placed where a dentist cannot be procured, to neglect acquiring a talent so easy and useful.

"Some dentists pull teeth directly out by a strong forceps made for the purpose; others use a forceps in the manner of the tooth-key, by resting one side of it on the gum as a fulcrum. In this case the resting side is formed like the bolster of a tooth-key. But much more in all cases depends on the dexterity of the operator than on the form of the instrument.

"*Steel trusses for ruptures* are one of the blessings to suffering humanity which modern ingenuity has supplied. From the unhealthy employments of some men in society, and the dissipation or unnatural modes of living of others, debilitated constitutions are frequent, and are often transmitted to offspring. One of the lamentable effects is that weakness of the sides of cavities in particular points, which allows the protrusion under the skin of the living parts from within. The occurrence is called hernia, or rupture: the most common hernia is that of the intestines, through the groins.

"Formerly this occurrence disabled for life. A man who had it was discharged from the army or navy; he could not ride on horseback, or take usual exercise; he could not lift a weight; and, in a word, he was often a miserable burden to himself and others. Now, by fitting the pad of a good steel truss to the part, the rupture is restrained permanently, and as perfectly as it could be for a moment by the hand of a skilful surgeon. The truss is put on and off with almost as little reflection or trouble as a part of the ordinary dress, and the man becomes again as fit for all the duties of life as if he were without his ailment.

"The old form of the steel truss was that of a half or three-quarter hoop, so bent and tempered that, when put upon the patient, one end with a pad upon it pressed inwards with a given force on the opening by which the rupture protruded. The defects in this kind of truss are, that it is difficult to make it fit exactly; it is rather troublesome to put on and off; and it bends or presses disagreeably all round the body.

"The other kind, which is free from these defects, consists of a little more than half a hoop, with a pad at each end: one of the pads supports the weakness, and the other rests upon the centre of the back, to bear all the strain there, while the hoop itself hangs loosely on the side of the body. This truss almost falls into its place of itself, and needs no fastenings: the same truss fits all persons of one size, whatever their shape; and the strength may be adjusted by changing the number of the springs of which the hoop consists.

"*Tourniquets, crutches, splints, &c. &c.* are so simple in all respects as not to merit special notice here.

"This section contains some of the reflections which occur to a person familiar with mechanical philosophy, in contemplating the human skeleton, and the more complete such a person's knowledge is of anatomy, physiology, surgery, and medicine, the more numerous will be the professional objects on which this philosophy will shed a light dissipating doubt and error. The author has not entered into more minute detail, because it would have been encroaching upon the office of those who teach particular departments; and because he thinks that any one who is not enabled by the examples here given to make the applications of the general laws to all possible cases, may account the study of the healing art unsuited to the faculties with which he is endowed." (P. 220.)

A section is devoted to the doctrines of Fluidity in relation to Animals, in which the phenomena attending the circulation of the blood is first described. Into this discussion it is not our intention to enter: we may merely remark, that our author attributes little effect to the "suction power" of the heart or chest in effecting the circulation, while he regards the action of the capillaries as of primary importance. His arguments against the doctrine which attributes the return of the blood through the veins to atmospheric pressure, will be found in our notice of Mr. SEARLE's answer to Dr. BARRY, in the Number of this Journal for June.*

The Circulation through the Head is ill understood by many, notwithstanding the interesting papers of Dr. KELLY and Dr. CARSON on this subject. In the work before us, it is well explained in the following passage:

"The head may be considered as an air-tight vessel or cavity of bone, containing chiefly brain and blood, and of which the openings are in communication with the blood-vessels leading to and from the heart. The atmospheric pressure, therefore, always keeps the head full, as it keeps the top of a syphon full; and, because the substance of the brain does not sensibly change its bulk by any ordinary degree of pressure, there must always be the same quantity of blood in the head, how much soever the quantity may vary in the body generally. Regard to this important truth, made intelligible by the discovery of the true nature of atmospheric pressure, enables us to explain many hitherto obscure facts, both in health and in disease.

"If from any cause the arteries in the head become too full of blood, in the same proportion the veins must become too empty; or if the veins be too full, the arteries must be too empty; and in either case the circulation in the head will be in a corresponding degree impeded, because, when any confined channel is narrowed or diminished in one part, the current throughout the whole is slackened. Now, as insensibility supervenes when the supply of fresh blood to the brain is interrupted, and death follows if the interruption continue long, it seems evident that, in many of the cases of apoplexy, where, on inspection, there is found nothing but a fulness of the arterial or of the venous system of the head, death has happened merely because the circulation was arrested in this way. In other parts of the body not circumstanced like the brain, such unequal distribution of blood happens with perfect impunity to the individual.

"Simple increase of pressure produced by the blood on the brain, provided the proper balance exist between the quantity in veins and arteries, has no injurious effect. This is proved by the descent of a person in the diving-bell, where, at thirty-four feet under the surface of the water, the body is bearing an additional pressure of fifteen pounds on the square inch, which affects the

* We likewise beg to refer to the paper of Dr. CARSON in the preceding, and of Dr. ARNOTT in the present, Number of this Journal.

brain through the blood-vessels, just as much as any other part.—On the other hand, when a man climbs a mountain, or ascends in a balloon, the brain is less pressed than usual; but, the proper balance of artery and vein being maintained, no inconvenience is felt from this cause. The inhabitants of some of the valleys among the Andes are as far above the sea as they would be at the top of Mont Blanc, but they enjoy good health.

"As the box of the cranium encloses the brain so as to leave no vacant space, it is evident that, when the heart injects blood with unusual violence, the strain at first is chiefly borne by the cranium itself, and not by the coats of the blood-vessels. Hence the arteries of the brain are not nearly so strong as those of other parts of the body.

"The veins of the brain are also peculiar. Common veins would collapse by any sudden tension of the arteries, and, if they did, insensibility or death would ensue, on account of the consequent stoppage of the circulation. The chief channels, therefore, for the refluent blood, instead of being common compressible veins, are what have been called *sinuses,* or grooves in the bone itself, with exceedingly strong membranous coverings, supported so powerfully that the whole become in strength little inferior to complete channels of bone. The singular deviation in the structure of the cerebral veins from what is found elsewhere, and without which animal existence could not have continued, is one of those particulars which powerfully affect the contemplative mind, as proofs of the design which has planned this glorious universe.

"From not adverting sufficiently to the circumstance which we are now explaining, of the cranium being a vessel which is always full, and will only hold a certain quantity, misconception has prevailed among medical men with respect to many of the affections of the brain.

"It has been said, for instance, that the substance of the brain cannot bear pressure with impunity, for that stupor immediately follows it, however produced. Now the truth is, that pressure produces stupor only when it stops the circulation. In wounds with loss of a large piece of the cranium, the brain will bear very rough handling, because, if compressed in one part, it may extend in another, and the circulation remain free. But if the wound be small, pressure made through it instantly affects the whole brain, and the blood from below is prevented from entering.—Let one reflect for an instant on what happens to the head of the child during parturition, how often it comes into the world elongated and bent, almost as if it were of soft clay, and for the time hideous: yet the child lives and thrives as well as if nothing of the kind had happened. The reason is, that the fœtal skull is soft, and pressure in one part is relieved by a corresponding bulging or extension in another, and the blood is not expelled.

"Water in the head, again, is said to kill by this fatal pressure on the tender brain; but in reality it kills by mechanically arresting the circulation. Accordingly we see that, where the fontanelle still remains open, or where the sutures or joinings of the skull yield, water may accumulate to a great degree without causing disturbance.

"A tumor in the brain, which would be of no consequence if the brain were unconfined, soon becomes fatal by checking the supply of blood.

"If the substance of the brain at all increase and diminish in bulk, like muscles, &c. in the body below, all such changes must produce a considerable effect on the cerebral circulation and functions." (P. 526.)

The only other part of this subject to which we shall allude is contained in the following proposed method of producing an effect analogous to venesection, without the actual abstraction of blood.

"A great proportion of dangerous diseases involve in their nature inflammation of some vital organ; and inflammation consists chiefly, as already stated, of a gorging or over-distention of the capillary vessels in the part. The nature of the capillaries, again, is such that, when not constantly filled by the pressure of the heart behind them, they gradually empty themselves towards the veins by their own action,—as is seen in the disappearance of a mortal inflammation soon after the death of the person; in the fact of the arteries being emptied of blood after breathing ceases, &c. Now, ever since medicine deserved the name of an art, practitioners have accounted the lancet their sheet-anchor in inflammatory disease; but it is only in late times, after the circulation of the blood was understood, that they have known the rationale of the remedy, —viz. that it acts by diminishing vascular tension, and thence the action of the heart, and so allowing the small vessels to empty themselves by their own force, and to recover sufficiently to resist the return of an excessive load. It is still more lately that they have known and understood how much more suddenly and completely the disease is cured by abstraction of a small quantity of blood *so rapidly* as to produce fainting, than of a much larger quantity *so slowly* that only weakness follows. Judicious treatment now cures inflammation more certainly and better than was done formerly, yet with much smaller loss of the precious blood, and less danger of those diseases of weakness, or of a complete breaking-up of the constitution, which often follow great depletion. To induce faintness, *large* openings into the veins are made, and often into two veins at once, and the patient is kept in the upright attitude. Often thus an inflamed eye, which was as red as scarlet before bleeding, in a few minutes is rendered nearly of the natural appearance; and most intense inflammations of the brain, lungs, bowels, &c. yield in the same manner. In all these cases the faintness seems to be equally efficacious, whether it happens after the loss of ten ounces of blood or of fifty; or, even as sometimes occurs, without bleeding at all, after merely tying the arm in preparation.

"Reflection upon these circumstances led the author to think that, in certain cases, the beneficial effects of blood-letting might be attainable by the very simple means of extensive *dry-cupping;* that is to say, by merely diminishing the atmospherical pressure on a considerable part of the body, on the principle of the cupping glass used very gently, and thus suddenly removing for a time from about the heart a quantity of blood sufficient to produce faintness. The results of trial have been such as to give great interest to the inquiry, and the author's first leisure will be devoted to the prosecution of it.—An air-tight case of copper or tin plate being put upon a limb, and closed by tying its leathern collar round the limb with a garter, on part of the air being then extracted by a suitable syringe, in an instant the vessels all over the limb become greatly distended with blood; and, as the blood is suddenly taken from the centre of the body, faintness is produced, just as by bleeding from a vein. The excess of blood may be retained in the limb as long as desired, for the circulation in it is not impeded. To produce a powerful effect with a slight diminution of pressure, more than one limb must be operated upon at the same time.

"An instrument resembling the contrivance now described was proposed, about twenty years ago, by a non-professional person, as a means of drawing all sorts of diseases out of the body through the pores of the skin. He enclosed a leg in an air-tight case, he then admitted steam to heat the limb, and relax the pores of the skin, as he said, and then he worked an air-pump to draw out the disease. He called the engine the *air-pump vapour-bath.* In various cases, where its true action was desirable, although not understood by the proposer, nor judiciously managed, it proved beneficial." (P. 533.)

Under the heads of Respiration and Voice, the physiology of these functions is explained in the same simple yet scientific manner which characterises the other parts of the work; but they are both too voluminous and too elementary for quotation.

The doctrines of Fluidity, illustrating and illustrated by certain phenomena of digestion, is likewise an interesting section. The following observations may be of use to those who happen to be called upon to attend persons who have swallowed poison.

"As a pump may not always be procurable when the occasion for it arises, it is important for the profession to be aware that a simple tube will, in many cases, answer the purpose as well, if not better. If the tube be introduced, and the body of the patient be so placed that the tube forms a downward channel from the stomach, all fluid matter will escape from the stomach by it, as water escapes from a funnel by its pipe; and if the outer end of the tube be kept immersed in liquid, there will be during the discharge a pumping action of considerable power. On changing the posture of the body, water may be poured in through the same tube to wash the stomach. Such a tube might be rendered a syphon, if desired, the necessary preliminary suction being made by the mouth through an intervening vessel.

"But there is still an easier mode than either of those already described of dislodging poison from a torpid stomach, and the author has used it successfully,—viz. merely to place the patient so that his mouth shall be considerably lower than the stomach,—as when a man's body is lying on a sofa, and his face is brought near the floor,—and then to press on the stomach with the hand. The cardiac orifice opens readily in such a case, and the stomach empties itself like any other inverted vessel.

"Useful as the pump may prove in evacuating the stomach upon occasions, its more ancient office of injecting enema is still the most important: and recent experience seems to shew that the injection of fluid *per anum* may become a remedy of more extensive utility than had yet been suspected. From an erroneous opinion that what has been called the valve of the cœcum acts as a perfect valve, allowing passage forwards only, few practitioners have ventured to order much liquid to be injected, for fear of over-stretching or bursting the lower part of the gut; and the possibility of relieving disease above the supposed valve had scarcely been contemplated. It is now ascertained, however, that fluid may be safely injected even until it reach the stomach. Perhaps few, if any, cases of obstruction of bowels could resist the gentle force of penetrating water; and, if so, a mechanical remedy of certain effect may be substituted for the drastic purgatives and pernicious bleedings now used, and often used in vain.

"From what has been said above of the abdomen and the intestinal canal, it appears that an injection tends to spread itself evenly over the whole. This may be rendered obvious to sight by throwing a sheep's intestine, recently extracted, into a bucket of water, and then pumping water in at one end. A stream will issue strongly at the other end, although several feet distant, almost immediately, and without any intermediate part having become sensibly tense. Of course, in the living body, where there may be spasm or obstruction, the liquid must be thrown in against resistance very gradually.

"That case is called introsusception of the bowel where an upper portion falls, or is received into a portion below, and the receiving part then, mistaking the received for descending food, holds it fast. This occurrence forms a complete obstruction, and generally proves fatal. Many infants, with irritable bowels, die of it. Now a copious enema, such as we have described above, is almost a certain cure. The liquid advances until it comes to the part where the portion of gut has been swallowed by gut below; and, as it cannot pass without pushing the introsuscepted portion back to liberty, it effects the cure." (P. 578.)

Another striking illustration of the advantages which may be derived from the application of philosophical principles to the practical part of surgery, is given in speaking of the Pelvic Apparatus. This consists in the description of some contrivances resorted to by the author's brother, Dr. James Arnott, and subsequently adopted by Dr. Ducamp, who dishonestly gave them as his own.

"The objects aimed at by the new means were, to ascertain the exact condition of the diseased canal,—to facilitate the passing of instruments in cases of difficulty,—and to effect a permanent cure. The following seven means may be particularised.

"1st. *An examining sound:* being a bougie with the point formed of a softer tenacious material, in which fibres of cotton or silk are mixed to prevent any portion from being broken off or detached during use. This sound pressed against the obstruction takes a correct impression of its anterior face, and shows the magnitude and exact position of the remaining opening.

"2d. *An expanding or dilator sound,* which is a small tube with a dilatable button at its extremity. The button consists of a little bag, empty while it is passing through the stricture, and filled with fluid when it has reached beyond. It readily discovers any other strictures beyond the first, and the state of each.

"3d. *A conducting canula,* or tube, open at both ends. It is passed down to the stricture, for the purpose of supporting and directing small bougies seeking entrance through very narrow strictures, or of guarding the caustic bougie in its approach to the place of its action.

"4th. In cases where the attempt to open the passage has failed by all common means, a conducting tube is first introduced, and through it six or more small bougies are then passed side by side, so as to probe the whole face of the stricture at the same time. It is thus scarcely possible that the opening should not be found.

"5th. Were even this means to fail, the conducting tube may be filled with water, under any degree of pressure, which water will either open the passage for the small bougies, or will itself act as the sharpest and most insinuating of all instruments. The stricture, by whichever means opened, will then allow the urine to escape. As patients might fear that water forced towards a bladder already too full would only increase the evil, J. Arnott waited for more numerous proofs of the utility and safety of the practice, before strongly recommending it: Dr. Amussat, of Paris, has lately published a statement of numerous cases of retention thus relieved.

6th. *A dilator* for widening the stricture, after a small instrument can be passed through it: it is intended as a substitute for the bougies and sounds of former times. The chief objections to these are, the painful friction, the danger of making false passages, the tediousness and imperfection of the cure, and that they cannot dilate any part of the canal beyond the size of its orifice, which during health is the narrowest part of it.

"The dilator consists of a tube of thin membrane introduced empty into the stricture, on a ball-pointed wire, and then filled with fluid by a syringe, so as to dilate with any degree of force, from the mere filling of the part to the strain of the hydrostatic press, which will tear the strongest texture that disease can form. The dilating tube is about two inches long, and its near end is fixed to the point of a small catheter, through which the distending fluid is injected. The tube is formed of thin silk riband of various sizes, with the edges joined. It is lined with prepared gut of the cat or dog, which is almost as thin as gold-beater's skin, although very strong and water-tight; and it is covered with the same, to give the smoothest and softest possible external surface. When complete and enclosing the blunt wire, it is less bulky than the bougie which would be required for the same case. Thus, it passes easily; it cannot tear the canal, or make false passages; it can enter through a small orifice, and dilate beyond to any desired extent; and its greatest advantage is, that by opening so as to follow the yielding of the stricture, it can affect at one application what only a succession of hard bougies, with long treatment, could accomplish. In one day it has often removed disease which had resisted other means for months, or even years." (P. 588.)

With this we close our analysis. It is but justice to the author to observe, that, while he directs the attention of the profession to the importance of physics, he guards the reader against allowing his mind to dwell too exclusively upon mechanical principles, seeing that, in the animal body, they are associated in their operations with the laws of chemistry and of life.

In conclusion, we recommend the work as calculated, by its simplicity, to lead on the student to the cultivation of an interesting and instructive subject, in general too much neglected; while it will prove refreshing to those who, having at some former period studied the principles of the science, have suffered their impressions to fade amid the practical pursuits of their profession.

*Lectures on the Operative Surgery of the Eye; or, an Historical and Critical Inquiry into the Methods recommended for the Cure of Cataract, for the Formation of an Artificial Pupil, &c. &c. &c. Containing a new Method of Operating for Cataract by Extraction, which obviates the Difficulties and Dangers hitherto attendant on that Operation: being the Substance of that part of the Author's Course of Lectures on the Principles and Practice of Surgery which relates to the Operations on the Eye and its Appendages.* By G. J. GUTHRIE, F.R.S. Surgeon to the Westminster Hospital, to the Royal Westminster Infirmary for Diseases of the Eye, Lecturer on Surgery, &c. &c. &c. *Second Edition, with seven Explanatory Plates.*—8vo. pp. 554. Burgess and Hill, London, 1827.

WE furnished our readers with so copious an analysis of the first edition of this valuable work, that we should have passed over the present edition with a very brief notice, if we did not discover in it many important additions that are worthy of attention.

At the commencement of the volume we find a Report of the Committee of Management, for the year 1826, of the Royal Westminster Infirmary for Diseases of the Eye, which bears ample testimony to the utility of the institution, and the attention and skill of the medical officers belonging to it. We may observe, *en passant*, that all persons labouring under diseases of the eye are admitted to the benefits of this charity without a letter of recommendation.

It is remarkable that, before the year 1817, no lectures were delivered in London on the diseases of the eye, unless, indeed, the concise observations which were made in the anatomical schools, on some of the operations, could be considered as such. The consequence necessarily was, that an intimate knowledge of the various complaints to which this organ was subject was not presumed to form part of the education of a surgeon. In order to remove some of the difficulties which impeded the progress of practitioners, and to render an acquaintance with these complaints more easily attainable by students, lectures on the anatomy and diseases of the eye were commenced by Dr. FORBES and Mr. GUTHRIE, shortly after they were appointed to perfo rm the duties of the establishment to which we have just alluded.

The most extensive benefits have resulted from this institution, not only by affording relief to numerous patients, but also by impartinginstruction to the many students, and even practitioners, who have had opportunities of witnessing the performance of the various operations connected with the eye, and the best modes of treating ophthalmic diseases.

It is properly urged by Mr. Guthrie that "a knowledge of the diseases of the eye cannot be acquired from lectures alone, or by reading: it is necessary to study them by diligent examination, and an attentive observation of the individual who is the subject of them. This can only be done at an hospital or an infirmary, where the opportunities are numerous; but then a competent acquaintance with them may be obtained in a few months, and with very little comparative labour. The diseases are nearly all open to inspection, most of the changes are under observation, and the effects of disease may almost always be predicted, if they cannot be prevented." (*Preface*, p. vii.)

It is, of course, not our intention to pass formally through each chapter of the second edition of a work which has before occupied so much of our attention. We shall merely touch upon those points in which Mr. Guthrie has made additions to his former statements.

Upon the subject of *inversion of the eyelids*, our author remarks that it has been supposed that there may be a shortening of the cartilage itself in these cases. He considers this to be impossible, and observes that he has never found it even softened, which SCARPA conjectures may sometimes occur. The practical observation of the author has led him to the following conclusions:

"1. That the derangement of the eyelashes, constituting trichiasis, is frequently the consequence of disease; but seldom or never arises from unnatural formation, or from an accidental or too luxuriant growth of the cilia.

"2. The relaxation of the integuments, or a partial paralysis of the levator palpebræ, in a natural and otherwise healthy state of the eye and eyelids, are not primarily concerned in the formation of entropium, and never alone give rise to it; although, if other derangement take place, they may (by removing some power of resistance to it) assist in its more complete formation.

"3. That, in a general or complete inversion of the edge of the tarsus of the upper lid, the swelling of the external parts, and the spasmodic action of the orbicularis palpebrarum, first tend to the formation of the disease, which is completely established by the contraction of the angles of the lids, and the accidental inversion of such hairs as become stiff and matted by the vitiated discharge from the meibomian glands and conjunctiva.

"4. That the change in the curvature of the lid takes place principally from the contraction of the angles, whilst under the influence of the orbicularis, and not from the contraction of the conjunctiva, corresponding to the superior broad ligaments, which supports the tarsus, and maintains the shape of the upper eyelid." (P. 15.)

These conclusions, of course, do not refer to such partial cases of trichiasis as result from the formation of a cicatrix, tumor, or other direct and obvious cause.

But little alteration has been suggested in the mode of operating for trichiasis since the days of Hippocrates. After a review of the peculiar manner in which different operators proceed for the relief of this distressing complaint, Mr. Guthrie recommends the following as equal to the cure of the worst cases.

"The head being properly supported, the eyelids are to be gently separated; the patient is to be desired to refrain from making any effort whatsoever, and the surgeon is to wait until he sees that the lids are perfectly quiescent. A small narrow knife, or one blade of a blunt-pointed scissors, is then to be introduced close to the external angle, and a perpendicular incision made, of from a quarter to half an inch in extent, or of a sufficient length to render the eyelid quite free; the quiescent state of the lids, and especially of the orbicularis muscle, enabling the surgeon to cut closer to the angle than he otherwise could do, and thus to divide the ligament, or at least the extremity of the cartilage. Another incision is to be made, in a similar way, at the inner angle: this should not include the punctum lachrymale, but be external to it, as I have never found it necessary to divide it; for, although the tears may continue to pass through the lateral canal into the sac, when the punctum has been included in the incision, they do not do so with equal freedom, and there is some observable deformity. The length to which the perpendicular incisions at both angles ought to extend, must now be decided upon by the appearance of the part: they must be continued, if necessary, by repeated touches with the scissors, until that part of the eyelid containing the tarsal cartilage is perfectly free, and is evidently not acted upon by the fibres of the orbicularis muscle, which lie upon it. This frequently causes the incisions, and especially the internal one, to be longer than is usually supposed to be necessary. The part included in the incisions is now to be completely everted, and retained by the forefinger of the operator's left hand against the brow of the patient; when, if any lateral attachment be observed, acting upon, and drawing, or confining the lid, it is to be divided, which is in fact still elongating the incision. On letting the eyelid fall on the eye, the edge of the tarsus and the hairs will frequently appear in their natural situation, in consequence of the relaxation of the angles, which bound them down; but, if the tarsal cartilage has become altered in its curvature, this will be immediately perceived: it will turn inwards at its ciliary edge, and be completely bent at its extremities, more especially at the inner one, where it is more powerfully acted upon by the musculus ciliaris. On desiring the patient to raise the lid, he readily attempts it, but the action of the levator, in such cases of vicious curvature, causes the cartilage partly to resume its situation; and, on examination, the curve will be observed to be so permanently vicious for about an eighth of an inch at each extremity, and especially at the inner, that it cannot be easily induced to resume its actual situation. When this is the case, the cartilage is to be divided exactly at the place where it is bent, in its length, and in a direction at a right angle with the perpendicular incision. The portion thus slit is only connected with the common integuments of the eyelid; and, although this incision does not exceed one-eighth of an inch at both extremities, and in general is only necessary at the inner, it enables the surgeon more readily to remove the altered curvature of the part, Mr. Crampton recommends that the two inner points of the perpendicular incisions should be now united by the subsection of Ætius and Paulus Ægineta,—that is, by a division of the conjunctiva, which he supposes to be in a contracted state; but I have always found this division to be useless, and even injurious. It is useless, because it has no influence on the part, for a simple division of that kind unites or fills up in a very short time; and it is injurious, because, if made across the cartilage, it is apt to form a ridge or cicatrix which irritates the eye, and of which, among others, I have seen one particular and incurable instance. The operation being thus far accomplished, a fold of skin is to be cut away from that part of the eyelid included between the incisions; three or four ligatures are then to be introduced, and the divided parts, from which the fold has been removed, are to be neatly brought together by the ligatures, each of which ought to be twisted, and then fastened to the forehead by several short slips of sticking plaster, the ends being turned over the plasters near the hair, and retained in that situation, to prevent their slipping. In raising the fold of skin, care should be taken to do it regularly with the fingers: it is also essential to the success of this part of the operation that it be done as close as possible to the margin of the eyelid. It may then be grasped by the forceps of Beer, which have transverse pieces, slightly curved for the purpose at their extremities, and close with a spring. The piece thus included, which need not be large, may be cut away at one or more strokes of a large pair of curved or straight scissors. The ligatures should be inserted first at each angle: and, when the vicious curvature is considerable, I not only pass it through the skin, but take care that the internal one shall include, at its lower part, the outer edge of the margin of the eyelid, which, from its firmness, retains that ligature much longer than those which are passed through the skin only, and tends to prevent the possibility of a relapse. The ligatures thus placed are to be equally drawn up on the forehead, until the eyelid is completely everted, when they are to be fastened as directed. In order to prevent any attempt at union but by granulation, or a filling up of the incision, the edges are to be slightly touched with the sulphas cupri; the eye and eyelids are now to be carefully cleansed; a piece of lint, spread with the unguentum cetacei, is to be placed upon them; a small compress is to be put under the edge of the eye-bone and orbit; a retaining bandage covers the whole, and completes the different steps of the operation. When the disease is not considered of an inveterate nature, the ligatures may be introduced through the fold of skin, without cutting any portion away. They give little additional pain, and are infinitely more effectual than any other suspensorium with which I am acquainted, but they ought to be assisted by strips of adhesive plaster placed between them. The operation, accomplished with all the care I have described, will still fail, if equal attention be not daily paid to the subsequent dressing, on which, indeed, more depends than on the operation itself: so much, indeed, that I am disposed to consider inattention to it the most certain cause of failure. In more than one hundred operations that I have performed, I have never known inflammation or other bad consequences ensue. The patient is to be kept perfectly quiet until the next morning, when the bandage and lint are to be removed, the eye carefully fomented and cleansed with warm water, and the dressings replaced. On the second day, great attention must be paid that the ligatures keep the lid sufficiently raised; and, if any union has taken place by adhesion at the angles of the incisions, it must be broken through with the probe. On the third day, the plasters, attaching the ligatures to the forehead, will in general require to be exchanged. The ligatures themselves must be supported by straps of plaster placed vertically between them; the edges of the incisions should again be touched with the sulphas cupri, or separated by the probe. The great art of the cure now consists in causing the incisions to be filled up by granulations only, so that the eyelid may be lengthened as much as possible, which can only be effected by a continuance of the means indicated. In a few days more, and especially by the continued elevation of the lid, the ligatures cut their way out, during which period the eyelid is gradually lowered, and, by the time the incisions have filled up, it will have resumed its natural situation, and the cure will be completed. There will, in general, be an indentation in the ciliary margin of the tarsus, where the incisions were made, but this is sometimes scarcely or not at all perceptible, and never detrimental. If the patient should have kept the lids closed at the commencement of the operation by the action of the orbicularis, the first incision may not have been made as close as might be desired to the angle of the lids; and in this case a hair or two at that part may not afterwards obtain quite its or their original direction, and cause some inconvenience; but this does not often happen, as these hairs are usually carried clear of the eye." (P. 31.)

Relaxation of the upper eyelid is not an unfrequent complaint, and always is a very troublesome one. Mr. Guthrie relates several instances in which it has been successfully treated, and in which it was dependent upon different causes.

*Staphyloma* is a consequence of acute inflammation, and assumes various appearances, all bearing a general resemblance to each other. In the infant or very young person, the cornea seldom sloughs so completely as in the adult, and it also possesses in them a power of reproduction which causes it to resemble in thickness, although not in transparency, the original state of the cornea.

"The gradual projection of the cornea is supposed to depend on the pressure or impulse given by the continuance of the secretion of the aqueous humor posterior to the iris; whilst the attendant absorption of it is diminished in consequence of the abolition of the anterior chamber by the adhesion of the iris to the cornea. This supposition, which appears at first to be plausible, admits, however, of considerable doubt; for we seldom find that, after the staphyloma has attained a certain size, it continues to increase, which it ought to do if the cause remained in activity. The desire of obtaining relief does not arise from the pain or inconvenience caused by the projection, further than that the inability to close the lids gives rise to irritation, keeps up chronic inflammation, and therefore causes pain as well as deformity. The operations now in use for its cure do not, in many instances, destroy the secreting apparatus, which they ought to do on this presumed hypothesis, whilst they certainly tend to diminish the absorbing surface: I therefore am disposed to believe that nature is in general capable of regulating the secretion according to the absorption, unless when some particular impulse is given to the former, as in the commencement of the formation of a staphyloma, or, as frequently takes place at a subsequent period, when the eye becomes irritable from any cause whatsoever which may give rise to it, and that the disease takes place in consequence of increased secretion, rather than from deficient absorption; nothing being more common than to see eyes in which the anterior chamber seems to be obliterated by the iris lying directly against the cornea, without any apparent attempt at the formation of a staphyloma.

"The cure which nature endeavours to effect of this disease is by thinning one particular part until at last it bursts, and the aqueous humor is evacuated; but this bursting has been caused by progressive absorption, which, on that event taking place, if not at an antecedent period, becomes the ulcerative; the ulcer remains for several days, a drain is established, inflammation is excited, under which, in my opinion, an alteration takes place in the actions of the secretory apparatus, by which the secretion is diminished. The eye becomes smaller, the ulcer heals, but the eye still decreases in size; showing that the absorbent are superior to the increasing or secreting powers. If the ulcer and inflammation be more extensive, suppuration takes place internally, and the eye sinks.

"The manner of accounting for the formation of a conical total staphyloma appears to me to be also defective; for, in the disease which is called conical cornea, every part except the cornea seems to be in a state of health. In this disease the cornea loses its spherical appearance as a segment of a circle, and assumes the form of a pointed cone, from the apex of which the light is reflected so powerfully as to present to a spectator the appearance of a luminous spot, which when once seen can never be mistaken. I have two cases of this kind under my care at the infirmary, one in a young, the other in a middle-aged man: both eyes are affected in each, without any other appearance of disease save that of a very slight increase of vascularity. The cornea in such cases is decidedly thinner than usual, and, if irritation takes place upon it, the apex of the cone becomes opaque, and causes it to represent in shape a conical staphyloma. The cause of these two complaints cannot, then, be so essentially different as the German hypothesis would lead us to believe. The alteration of shape is incurable, but the defect of sight may be partially relieved by using a deep concave glass; which appears to me to answer better than an operation for the destruction or depression of the lens, which may not be, and has not always been, successful when attempted in such cases." (P. 181.)

Mr. Guthrie is of opinion that a partial staphyloma is always the result of ulceration of the cornea, through which the iris protrudes, and from which it obtains a thin covering. This becomes opaque, and of a pearly colour, and the iris can be seen firmly adhering to its internal surface; in which state it is merely a leucoma.

"The operations for the cure of staphyloma are effected in two ways,—one by the caustic, the other by the knife. The caustic is only applicable to cases of adult subjects, (very rarely of young ones,) in which the cornea forming the staphyloma is thin, and the sclerotica is nearly or quite free from disease. The operation by the knife is required in young or in old subjects, where the staphyloma is evidently thick and hard, and the anterior part of the eye in a more or less varicose state." (P. 184.)

Mr. Guthrie disapproves of the application of caustic, as recommended by Scarpa, in cases which originated during childhood, and have continued to manhood. In these instances the thickness of the cornea prevents the caustic from quickly penetrating into the anterior chamber, and causes it to produce a severe inflammation of the eye generally.

"The lunar caustic, which is the best of all the escharotics that have been recommended, should be reduced to a very fine point, and applied with a steady hand to the most prominent part of the staphyloma for the space of a minute, if the patient is sufficiently steady; and it ought to be repeated every second day until it has penetrated the cornea, which will in general be accomplished on the second application, if the first should not have been found sufficient. The caustic which has been dissolved during its use will do no harm; but, to be secure, the eye may be washed with a little warm water. On the separation of the slough, an irregular, small, but penetrating ulcer remains, through which the aqueous humour percolates until the opening closes, which it does in a few days, with a very moderate degree of inflammation and little pain. By this period the anterior part of the eye will have become so much flattened, that, although it prevented the closure of the eye-lids previously to the operation, it now offers no such obstacle, and the cure is equally certain and effectual, although less formidable, than if the operation had been done by the knife.

"When the staphyloma is firm and thick, as in young subjects, or where it has existed in an adult from childhood, its removal by the knife should always be preferred. The operation was well known to the ancients. Celsus describes it, and gives some very excellent precepts for its performance, which were subsequently neglected. The principle on which it ought to be done is well understood; the mode of doing it varies according to the state of the part." (P. 185.)

The operation of *extirpation of the eyeball* is sometimes necessary in consequence of a cancerous affection of its appendages, of fungus hæmatodes, and of tumors which implicate any part of it in such a manner as to render the removal of the whole unavoidable. Fungus hæmatodes of the eyeball is regarded by Mr. Guthrie as a fatal disease. He is not aware that the removal of the eye has succeeded in arresting the progress of the disease, when it has been so fully formed at the bottom of the eye as to show distinctly its nature. "The first sign of the disease is the loss of sight, soon followed by an appearance at the back of the posterior chamber, resembling burnished or polished iron, and accompanied by a dilatation and immobility of the pupil, by an enlargement of the vessels of the conjunctiva and sclerotica, and by pain in the eyeball. By degrees an amber or greenish coloured irregular spot is seen to arise from it, resembling a small mass of coagulable lymph, which may be mistaken for a partial opacity of the vitreous humour. This fungus gradually increases; and at this period the extirpation of the eye offers little or no chance of success, but will rather accelerate the death of the patient, as the disease has extended along the optic nerve, and will reappear after the eye has been removed." (P. 189.)

Mr. Guthrie has extirpated the ball of the eye five times, with little loss of blood, excepting in one case, in which the hemorrhage continued so long that it became necessary to fill the orbit with sponge to suppress it. The patient recovered without any bad symptom. The following is the manner in which Mr. G. performs this formidable operation.

"The patient being seated below the operator, or placed on his back, and his head being firmly secured, the upper eyelid should be raised by an assistant, whilst the surgeon passes the ligature, by the aid of a needle, through the cornea, or even the anterior part of the eye, when the needle should be cut off, and the upper lid allowed to fall. The surgeon should now cut through the external commissure down to the edge of the orbit, with a small, straight, but rather long and pointed scalpel, and then divide the conjunctiva and fat around the eyeball, beginning at the under part, to prevent the blood from above impeding the operation. The complete division of the outer angle of the lids down to the bone facilitates this part of the operation materially, as it allows the lower lid to be readily everted, and depressed by the forefinger of the left hand of the operator, thereby preventing its being injured, which is very likely to be done unless great care be taken to avoid it, by making the forefinger act as a director. The eyeball may then be drawn gently forwards or outwards, to offer a greater facility to the remaining steps of the operation, which consist in cutting deeply into the orbit with a pair of scissors, curved as directed on the back. In order to repress the hemorrhage, or rather to prevent its impeding the operation, an assistant should wash away the blood, by means of water injected into the wound by a syringe; and, when the eye is removed, the operator must carefully examine the orbit with the finger, to ascertain that no diseased part is left, and that the lachrymal gland has been removed. The hemorrhage is not to be dreaded; and, unless the patient is very weak, it is much better to let it cease under the use of cold water, than to fill the orbit with sponge or lint, neither of which can be of any use, unless they make compression, which is improper if it can be avoided." (P. 192.)

The author has dedicated a very long chapter to the discussion of *cataract;* and, from the manner in which every practical point connected with it is considered, it is very evident that his opinions are founded upon the substantial basis of extensive experience, assisted by much judgment and discrimination. Mr. G. observes, that there is "a very important distinction, which is frequently neglected, between idiopathic or constitutional, and local or accidental cataracts; not, as in the previous case, referring to the change which has taken place in the structure or appearance of the lens, or to the manner of operating, but to the comfort and happiness of the sufferer. The idiopathic or constitutional disease affects in general both eyes; the local or accidental being more often confined, under proper management, to the organ which has been injured either by external violence or active inflammation." (P. 199.)

We have no power of preventing the constitutional form of cataract. Our means are insufficient, from our being perfectly unaware of its approach, until our attention is drawn to it by the symptoms attendant on its progress.

We are compelled unwillingly to pass over many remarks upon the nature and progress of the different varieties of cataract, of much importance to the student of diseases of the eye.

Upon the *cure or removal of cataract*, Mr. Guthrie observes that—

"The cure of cataract, in its incipient stage, has been often considered practicable, and the methods or means of doing it, have been frequently recommended by different authors; which, whilst they induce us to place some confidence in an opinion so often repeated, lead us, after almost constant disappointment, to suppose they must be mistaken in the cases they considered as incipient cataracts, and that they were not constitutional or idiopathic, even if they were strictly local.

"There cannot be a doubt that persons who have had opacities behind the iris, perceptible to the eye of an observer, have been cured under a course of medicine of various kinds; but it is indisputable that these cases are very rare, even in the hands of those who place confidence in the opinion: and it is possible that the opacity had arisen from slight depositions in the capsule, the result of simple inflammation, rather than from any opacity of the crystalline itself; for we know that opacities, or rather a haziness of the capsule, caused by inflammation of the iris extending to it, may almost always be relieved under the treatment proper for the cure of iritis. That opacities in the capsule remain stationary for a series of years is well known; and I am disposed to believe that, where medicines have been given in such cases, they have obtained more credit than they deserved for arresting their progress, and that most of the cases reported to have been relieved or arrested have been of this kind; whilst those that have been cured have not been opacities of the lens itself, but of its capsule: or, if they were lenticular cataracts, they were caused by external violence, and disappeared in consequence of their dissolution and absorption in the aqueous humour, the opacity of the lens having been the result of a rupture of its capsule. Mr. Ware, who at one time supposed incipient cataracts might be cured by spirituous applications, and particularly the sulphuric ether, latterly abandoned the opinion; and it would seem, from a note in the third, that the cases he published in the first and second editions of his work on Cataract, and which followed an external injury, were of the latter description.

"When an injury is inflicted on the eye by any substance which penetrates the cornea, and opens the capsule of the lens without displacing it from its situation, it gives rise to the formation of a

2

capsulo-lenticular cataract, which is usually a soft one, and readily removed by absorption, provided the opening in the capsule does not close, which would put a stop to this process, or cause at least a change to take place in the nature and appearance of the cataract, rendering the capsule, in the generality of cases, harder, tougher, or leathery, or what is termed by Schmidt and Beer, siliquose. If the wound in the capsule should have been of greater extent, the capsule yields, and either shrinks or is itself absorbed, so as to leave the pupil perfectly clear, and in this way give rise to the opinion of a cataract having been cured by the external remedies applied to the eyelids or eye. Upon this fact the operation (the keratonyxis) through the cornea for the opening of the capsule, and the consequent removal of the opaque lens, was founded, and is liable, as will be hereafter explained, to the same difficulties; for if, in any way in which the operation be done, whether accidentally or purposely, the lens be displaced, it presses on the iris, as a large solid body, and frequently causes inflammation, closure of the pupil, and other deformity, which is often irremediable. When a cataract has been already formed, and from some accidental violence the capsule is ruptured, the cure may be completed in the same manner, and be attributed to the use of remedies which have been perfectly inert.

"In doubting then that the removal of a well-formed opacity of the lens can be accomplished by external applications or internal remedies, it is also proper to confirm the opinion that opacities of the capsule may be sometimes arrested, frequently diminished, and sometimes removed, by a due appropriation of these means; but even in these cases, I believe, it is principally such opacities as are the consequence of common inflammation that are amenable to their influence, or the true idiopathic cataract in an incipient state." (P. 260.)

M. Gondret,* however, asserts that he has been able to remove opacities at an early period of their existence, by a combined internal and external treatment. In illustration of his opinion, he has adduced some cases, two of which Mr. Guthrie transcribes. The first case recorded by Gondret is not considered by our author to be one of pure cataract, and he observes that, if the others had been like it, the reputation of the practice for the cure of cataract could not have been maintained. Two of the cases, however, are deemed unobjectionable, and, "provided no mistake has been made, establish the fact of the possibility of removing an opacity of the lens without an operation. Gondret admits that, when the disease is fully established, the means recommended are not sufficient; but the precise state at which the removal of the

* Mémoire sur le Traitement de la Cataracte, by L. F. Gondret, &c.—Paris, 1825.

opacity is likely to be accomplished is not ascertained, and therefore the practice may be tried in all incipient cases."

The treatment adopted by Mr. Guthrie is described in the following extract:

"I have been in the habit of making caustic issues on the top of the head and on the temples for several years past, and of continuing them for months, as the students attending at the infirmary can testify, in cases resembling the first, and with considerable success. This treatment has been combined with galvanism, alteratives, and strict attention to the general health, and to any prevailing diathesis, such as gout, rheumatism, &c.; but I have never seen a distinct well-formed cataract removed by it. Patients have often improved in sight, their pains have ceased, the eyes have re-assumed a natural appearance, or the alterations of structure have not extended; and so far this may be considered a cure. I have several cases now under treatment, and I shall be very glad to find that a great extension of the practice, in cases of incipient cataract, will lead to a more favourable result than has hitherto been attained in this country. Admitting, as I do, that an operation on one eye is certainly capable in some instances of causing the removal of a commencing opacity in the other, it is reasonable to suppose that some means may yet be found of effecting this object in a certain and more satisfactory manner. Incipient cataracts ought not, therefore, to be left until the opacity is complete, but should be submitted to a treatment adapted to the age and habit of the patient." (P. 265.)

Our readers are probably aware that, in the publication of Mr. Stevenson upon the subject of cataract, it is contended that the removal of a cataract from one eye will prevent the formation of the disease in the other eye, or will cause its disappearance, if forming. Mr. Guthrie states that the opinion that the removal of a cataract in one eye would prevent the formation of an opacity in the other, does not belong to Mr. Stevenson. It is conceded, however, that Mr. S. has the merit of reviving the idea, which may be acted upon with advantage in some cases.

The doctrines of Mr. Stevenson upon some other points are thus canvassed by Mr. Guthrie:

"In regard to the second supposition, 'that the operation to effect this removal of the cataract may *always* be done by puncturing the lens, or the absorbent practice, to the rejection of the methods by extraction and depression,' there cannot be the slightest doubt of it being most unfounded: indeed, Mr. Stevenson must be aware that, when the cataract is a hard or indurated one, the absorbent practice will not only not succeed, but will in general be followed by the most unfortunate result. I shall therefore presume that he limits the expression to the earliest period at which the formation of a hard lenticular cataract can be perceived. I am not prepared to say that at this period the absorbent practice may not succeed; but this I know, that long before the hard lenticular cataract is completely formed, long before the patient has ceased to see objects at a few yards' distance, the lens is too hard to be broken up by the knife, and that at this period the operation by the absorbent practice is neither the safest nor the best. In cases of soft cataract, the absorbent practice is at all times the best; but on these points I beg to refer the reader to the following parts of this work, in which these subjects are particularly noticed.

"The third supposition, 'that the operation ought to be done at the earliest possible period of time after the opacity is fairly perceived to exist,' is the only one to which I can assent; for, if the operation is likely to be followed by success, and not to meet with an insuperable impediment from the peculiar nature of the cataract, it can only be at the particular period of commencement. The patient must then be made aware of a fact, that, from the moment the needle enters the capsule of the lens, the opacity of that lens will rapidly increase, and in a few hours will induce perfect blindness, which can only be removed by the absorption of the lens and a successful result of the operation. Surgeons must either forget that by far the greater number of cataracts have made considerable progress before they are discovered to exist; and in many cases the cataract of one eye has gone on nearly to blindness before it is discovered.

"I shall conclude by observing—

"1. That the particular operation recommended by Mr. Stevenson is inapplicable in by far the greater number of persons afflicted with cataract, as they are usually met with, and, when attempted, will in general be followed by an unfavourable result.

"2. That it is an excellent operation in all cases of soft or fluid cataract.

"3. That Mr. Stevenson is correct in maintaining that an operation in one eye is capable, in certain cases, of causing the removal of a commencing opacity in the other.

"4. That the kind of operation to be selected must depend on the nature of the cataract.

"5. That Mr. Stevenson's proposition of operating at the earliest possible period at which an opacity can be perceived, is deserving of a further and more extended trial." (P. 279.)

In order to avoid some of the difficulties attendant on the division of the cornea in the operation for cataract, Dr. F. Jager, of Vienna, has proposed a double knife, of ingenious construction. An account of this instrument is given in Loudon's "Inquiry into the principal Causes of the unsuccessful Termination of Extraction by the Cornea." Mr. Guthrie has not derived so much advantage from the use of this knife as he had been led to expect; but he confesses that he is not as yet competent to give a decided opinion.

In denying, however, to this instrument the merit which has been attributed to it, Mr. G. is willing to concede that of its being the foundation for the construction of another, which he considers to be more perfect, and to be capable not only of preventing nearly all the difficulties and dangers which occur in the extraction of the cataract, but of obviating them when they have occurred. A description and plate of this improved instrument are given.

There are seven plates appended to the volume, which are well engraved and coloured, and very clearly illustrate the peculiar appearances of various diseases of the eye.

We advise every practitioner who wishes to acquire information upon the subjects of which it treats to consult Mr. Guthrie's work, as it contains a greater mass of useful information connected with diseases of the eye than any other with which we are acquainted. The practical observations have been principally made at the Infirmary in Warwick-street, where we ourselves have had frequent opportunities of witnessing the great dexterity of Mr. Guthrie in the performance of various operations on the eye.

---

*Spinal Diseases, illustrated with Cases and Engravings; also an Inquiry into the Origin and Cure of Distorted Limbs.* By Ed. Harrison, M.D. F.R.A.S. ED.; formerly President of the Royal Medical and Royal Physical Societies of Edinburgh, &c. &c.

The profession of late have been supplied with numerous works on diseases of the spine, and yet, after all the elaborate investigation which the subject has undergone, there is perhaps no organ in the body with regard to which a greater discrepancy of opinion prevails as to the causes, treatment, and pathology of its diseases. We can only account for the different views which various authors have taken of the nature and causes on which spinal distortions depend, by supposing that each has his attention too exclusively fixed on some particular part of the column, or some particular circumstance connected with it. For our own parts, we believe that every structure entering into the formation of the spine, whether bone, ligament, cartilage, or muscle, may originate distortions separately and collectively, either from constitutional causes, local injury, particular positions, or habits of life.

Dr. Harrison is of opinion that by far the most frequent cause of spinal maladies is some morbid change or relaxation of the ligaments of the spine, though he does not deny that other tissues connected with the spine may likewise be primarily diseased. To the doctrine of muscular action as a source of spinal distortion, our author makes the following objections:

"Were it to be proved that in the extremities two sets of muscles are acting in constant opposition to each other, it would not follow in course that those in the trunk are similarly employed: indeed, there is no such provision, as far as I know, in the structure of the back. The muscles situated there are most of them too large to be concerned in forming the lateral or sigmoid flexions of the spine, without supposing them to produce a different action at one end, from what they do at the other. Moreover, such an admission is altogether improbable, and is in direct opposition to every principle of muscular motion in other parts of the body. We have strong muscles to raise and bend the trunk; but they proceed in unison with one another, and are never placed in opposition. These two motions, the principal ones of which the spine is susceptible, are not synchronous; they interchange with each other, and consequently do not interfere." (P. 14.)

It is pretty evident that these movements can have nothing to do with the production of the posterior or angular variety of curvature; and even in lateral, where they are supposed to be so influential, one would at first sight imagine that they would simply draw the spine either to one side or to the other, and not give a serpentine character to the curvature. Probably they are not the only agents, even in lateral distortion, but act in combination with other means which conspire to this end.

Dr. Harrison, speaking of the influence which a diseased condition of the spinal marrow has upon the viscera, remarks that

"The functions of all the internal viscera are, it is well known, severely affected in spinal complaints. We clearly perceive, from this circumstance, that a close and intimate connexion subsists between the cord and these different organs, through the medium of the great sympathetic. It may be safely affirmed that, wherever the spinal nerves are distributed, however distant from their origin, disease may be produced by a distempered condition of the column. In this consideration of the subject, which will, I doubt not, be fully confirmed by subsequent experience, the vertebral pillar constitutes, in a medical view, the most important organ of the human frame." (P. 88.)

From this it appears that our author attaches much importance to the spinal marrow and its coverings, when morbidly affected, and it is very certain that functional derangement, more particularly at the commencement of curvature, does often obtain to a very high degree; yet organic lesion seldom, if ever, is found to occur. It cannot be denied but that the internal viscera will adapt themselves to very great deformity of the spine, and the person so mis-shapen enjoy good health.

The following extract will convey to our readers the opinions of Dr. Harrison on the origin, nature, and situation of spinal diseases, in which he makes some allusions to the rather sharp criticisms which have been made, by some cotemporary writers, on the first and second chapters of the present work, which were originally published in this Journal, in November 1820, and February 1821.

"This inquiry is the more necessary, because the occurrence of vertebral luxations, independently of external violence, appears to be commonly denied. I shall therefore premise a few of the reasons which have led me to entertain the affirmative. The vertebral column is a pillar of complicated and delicate mechanism. It is flexible, to provide for the motions of the trunk; and strong, to support the incumbent weight. The former intention is secured by its jointed structure; the latter, by numerous ligaments of great strength. An organ thus constituted is liable to many disorders, from the discordant nature of its materials, and complexity of its functions.

"It will be seen, from a consideration of the preceding details, that I place all vertebral distortions in the spinal column. But, although I entertain this opinion of their nature and situation, I was never led to contend that they originate in the same tissue. On the contrary, I have always maintained that they commence in different portions, and require different methods of cure according to the parts in which they are implanted. Some invade the bones, others the intervertebral substance, others again the articular compresses. These several species are characterised by appropriate symptoms, and must be carefully distinguished from one another, because each of them requires a peculiar mode of treatment for its removal." (P. 99.)

And again—

"In my endeavour to trace the seat of spinal disorders to a new structure, and to exhibit them in a different point of view, I am aware that I have taken an adventurous flight, and one that has already exposed me to much prejudice and undeserved obloquy. Encouraged, however, by the consideration that every honest endeavour to relieve our afflicted fellow-creatures is entitled to a patient and candid investigation, I shall rely upon the liberality of my brethren, and no longer hesitate to explain my peculiar opinions. Though I have ventured distinctly to assert that spinal maladies and distortions are frequently situated in the ligaments, I would not, as I have already stated, be understood to insinuate that they are invariably confined to this fabric." (P. 102.)

There is no doubt in our minds that the spine partakes more immediately of the character of a jointed structure, and admits of greater mobility in consequence than is generally allowed; and we think it not unlikely that, in those cases where the bodies of the vertebræ, ligaments, or intervertebral substance, undergo a change from constitutional causes, such an elongation of all the connecting ligaments does take place, sufficient to give rise to a displacement which may be appropriately termed a subluxation: indeed, those extensive angular projections and obliquities cannot be accounted for on any other principle, particularly in the dorsal and cervical portions of the column.* It is generally thought that death must inevitably ensue from such a condition of the vertebræ pressing on the spinal marrow: we confess we cannot bring ourselves to think so, for it has been proved by dissection that the spinal marrow may be compressed, by the operation of slow and long-continued causes, into almost half its circular diameter, without any interruption taking place in its functions.

The injurious effects of posture, even in healthy people, in the various occupations of life, but particularly in delicate females in the higher circles of society, who are exposed by the present system of education to so many concurring circumstances which produce a debilitated condition of the frame, cannot be too generally known.

"A knowledge (says the author,) of the injurious effects of posture upon the spines of hardy labourers, cannot be too strongly impressed upon the minds of parents and teachers. I have already adverted to the over-anxiety to educate girls in the fascinating accomplishments of music, drawing, and dancing, in the prosecution of which they are confined in hot rooms, and forced into strained attitudes for hours together, and to the manner in which it has led, in different ways, to the most distressing consequences. If long perseverance in any habit be sufficient to produce distortion and deformity in the spinal arrangement of adult and athletic males, it will surely be much more likely to induce them in the sickly and pampered children of the affluent. Though various causes may be assigned for the increased prevalence of these complaints in our time, I am convinced that the relaxing effects of hot rooms, and a too-ardent pursuit after feminine accomplishments, are the principal ones." (P. 117.)

Dr. Harrison supposes that, although no direct anatomical connexions of the spinal nerves have been traced into the substance of the viscera, yet in his opinion, from their connexion with the great sympathetic, the nervous power of the spinal marrow is thus transmitted, and which accounts for the derangement of their several functions in the onset of vertebral

* We do not pledge ourselves as concurring in all the opinions expressed in this review: that which refers to *subluxation*, indeed, is in opposition to what we have stated on former occasions.—E.

distortions; and it would appear that, from the most early period of medical science, some of the important facts which make so brilliant an era in modern physiological investigations, were shrewdly anticipated by some of the sages of antiquity. Dr. H. states,

"In tracing the leading symptoms of spinal complaints, we shall find that they may be chiefly referred either to inordinate action of the muscles, or disturbance in the internal viscera, and the organs of sense. All are affected, although the disease prevails sometimes more in one system, sometimes in another.

"There is no difficulty in understanding the former. They derive all their nerves immediately from the spinal cord. Hence we can explain why the muscles are affected, when it, or the branches derived from that source, are irritated, compressed, and stretched, in the foramina vertebrarum, or in their progress to the muscles.

"Though we may not be able to trace the anatomical connexion of the spine and viscera, we nevertheless do not hesitate to infer its existence from the internal commotions observable in these complaints. The relation of the parts to one another is so constant and uniform, that, after hearing the details, I seldom find much difficulty in fixing upon the displaced vertebræ, even before I have made a personal examination. My deduction is founded upon an uniformity of symptoms, which appears to depend upon the connexion between certain portions of the spine and certain organs. Nor does our inability clearly to establish the intercourse, by following the nervous fibrils from one to the other, invalidate the principle. Many things are true which anatomy, notwithstanding its numerous improvements, has not yet been able to develope: indeed, few of our medical facts, or pathological principles, were first observed by the anatomist: we are indebted for both to the practical physicians, who first discovered them in their professional attendance upon the sick. My detection of symptoms, and their seat in the spinal column, was first drawn from the same source. Ever since my attention was fixed upon the subject, they have been so constantly observed in practice, that I have no hesitation in pointing them out as uniform in their appearance, and produced, in some way or other, by the distorted figure of the spinal column. It has happened to me so frequently to remove internal derangements by rectifying the vertebral pillar, that, when summoned to patients of this description, I do not hesitate to predict a complete cure, if the spinal distortion can be removed. Although anatomists may have hitherto failed in their efforts to prosecute nervous filaments from the spinal cord into the viscera, we must not from thence hastily conclude that the communication does not exist. We find little difficulty in following these nerves from the spinal cord into the very substance of the great sympathetic, or in pursuing other nerves issuing thence to the affected organs. In this way an intermediate connexion is found to obtain between the spinal column and internal parts, sufficient to explain the pathological deductions which have been formed. Were the relations even less apparent, we ought not on that account to deny the evidence of our senses, or the dictates of experience. Hippocrates informed us, more than two thousand years ago, that parts lose their feeling, while motion remains entire; and that at other times motion is destroyed, leaving the sensibility unimpaired. Rufus, the Ephesian, who lived about the end of the first Christian century, followed up these remarks, and endeavoured to explain them on anatomical principles. He paid great attention to the brain and nerves in his dissections of brutes. It was his opinion that some nerves perform the office of sensation, while others are confined entirely to motion. These facts were uniformly observed and admitted, although the rationale continued unknown through so many ages. At length the mystery has been revealed by the industry of anatomists; and it may be reserved for other investigators to demonstrate the connexion of the spine and viscera, by pointing out the intermediate links." (P. 281.)

Dr. Harrison's method of treatment in spinal distortions consists in the recumbent posture, frictions, regulated pressure, bandages, with steel or wooden shields applied over the projecting vertebræ, occasional extension, &c. Nine cases are related (some of which are illustrated by good engravings) successfully treated in this manner, and which appear to have been of the posterior or angular variety of curvature. We think that the third, fourth, and fifth show incontrovertibly that angular projections to a great degree are not always accompanied with ulceration or destruction of the bodies of any of the vertebræ, although they are generally supposed to depend upon such a condition of the spine, and treated by some of our most eminent surgeons, after the manner of Mr. Pott, by caustics and recumbent posture: indeed, some of the cases were thus managed, without the least benefit to the patient. We have no doubt that many cases of this sort of curvature exist without organic disease, and require a mode of treatment different from that usually adopted.

To most of our readers the opinions of Dr. Harrison must be familiar, from the series of papers which he published in this Journal; we have therefore thought it unnecessary to go much into detail on the present occasion.

We can recommend the work as containing various new views on spinal pathology, and as executed in a manner very creditable to the author.

