# Erratum

**Published:** 1827-09

**Authors:** 


ERRATUM.

In the present Number, in the running title to the Review of Dr. ARNOTT'S work, for "Elements of Physic," read "Elements of Physics."

